# Supplementary material for: replicAnt: a pipeline for generating annotated images of animals in complex environments using Unreal Engine
Source: Nat Commun. 2023 Nov 8;14:7195. doi: 10.1038/s41467-023-42898-9 (PMC10632501; doi:10.1038/s41467-023-42898-9)
Supplement: Supplementary file 1 — Supplementary Information [file 41467_2023_42898_MOESM1_ESM.pdf]

Detection and tracking datasets

Table 1 Benchmark *Atta* detection datasets

dataset	samples[1]	recording settings
base	1,000	camera = OpenCV OAK-D<br>fps = 25<br>shutter speed = 1/50 s<br>image size (px) = 1920 x 1080<br>individuals = 81<br>notes: well exposed, no motion blur,clean background
bright	1,000	camera = OpenCV OAK-D<br>fps = 25<br>shutter speed = 1/25 s<br>image size (px) = 1920 x 1080<br>individuals = 103<br>notes: highly overexposed, motion blur, clean background
dark	1,000	camera = OpenCV OAK-D<br>fps = 25<br>shutter speed = 1/200 s<br>image size (px) = 1920 x 1080<br>individuals = 65<br>notes: under exposed, extreme rolling shutter and compression artefacts, clean back-ground
noisy	1,000	camera = OpenCV OAK-D<br>fps = 25<br>shutter speed = 1/50 s<br>image size (px) = 1920 x 1080<br>individuals = 61<br>notes: well exposed, no motion blur, highly textured background
close	1,000	camera = OpenCV OAK-D<br>fps = 25<br>shutter speed = 1/50 s<br>image size (px) = 1920 x 1080<br>individuals = 36<br>notes: close-up top-down footage, slightly out-of-focus, clean background
all	6,000	camera = OpenCV OAK-D<br>fps = 25<br>image size (px) = 1920 x 1080<br>individuals = 36 to 103<br>notes: containing all real samples

[1] Using five-fold cross-validation, 20% of the samples are withheld during training.

Table 2 Synthetic *Atta* detection datasets

dataset	samples[1]	generator settings
multi ant<br>(S, M, L)	100 (S)<br>1,000 (M)<br>10,000 (L)	individuals = 100<br>scale variation = 33%<br>subject meshes = 3<br>image size (px) = 1024 x 1024<br>compression rand. = True<br>new terrain every = 15<br>new scatterers every = 10<br>new subject placement every = 5
single ant	10,000	individuals = 100<br>scale variation = 90%<br>subject meshes = 1<br>image size (px) = 1024 x 1024<br>compression rand. = True<br>new terrain every = 15<br>new scatterers every = 10<br>new subject placement every = 5

[1] Using five-fold cross-validation, 20% of the samples are withheld during training.

Table 3 Augmented / mixed *Atta* detection datasets

dataset	samples[1]	recording settings
base (augmented)	1,000	camera = OpenCV OAK-D<br>fps = 25<br>shutter speed = 1/50 s<br>image size (px) = 1920 x 1080<br>individuals = 81<br>notes: background replacement of base dataset using thresholding and randomly drawn ImageNet 2019 samples
bs10 − 10 real /1 synth<br>bs100 − 1 real / 1 synth<br>bs1000 − 1 real / 10 synth<br>sb5 − 1 real / 50 synth<br>sb1 − 1 real / 100 synth	1,100<br>2,000<br>11,000<br>10,500<br>10,100	notes: combining the (synthetic) multi-ant dataset with the (real) base dataset in various ratios

[1] Using five-fold cross-validation, 20% of the samples are withheld during training.

Table 4 *Gnathamitermes* detection datasets

data type	dataset	samples[1]	generator settings
synthetic	termites S	10,000	individuals = 100<br>scale variation = 50%<br>subject meshes = 2<br>image size (px) = 1024 x 1024<br>compression rand. = True<br>new terrain every = 15<br>new scatterers every = 10<br>new subject placement every = 1
real	termites R	10,000	camera = Nikon D810, 105 mm Nikkor lens<br>fps = 30<br>shutter speed = 1/200 s<br>ISO = 200<br>image size (px) = 3840 x 2160<br>individuals = 76<br>notes: filmed in the dessert, without controlled lighting conditions, daylight, highly textured background

[1] Using five-fold cross-validation, 20% of the samples are withheld during training.

Table 5 Case-specific detection performance (AP) of YOLOv4 (in *Atta*)

data type	dataset	samples[1]	base	bright	close	dark	noisy	mAP[2]
real	all	6,000	0.9999	0.9999	0.8535	0.9999	0.9999	0.9707[3]
real	base	1,000	0.9999	**0.9873**	0.6611	0.7701	0.8317	0.8125
	base (aug[4])	1,000	0.9925	0.9327	0.7273	0.9371	0.8358	0.8582
	bright	1,000	**0.9628**	0.9999	0.3014	0.2521	0.5350	0.5128
	close	1,000	0.8422	0.4547	0.8337	0.4025	0.6366	0.5840
	dark	1,000	0.9021	0.8610	0.8146	0.9999	0.8379	0.8539
	noisy	1,000	0.9557	0.9613	0.6904	0.9062	0.9999	0.8784
synthetic	multi ant (S)	100	0.7718	0.3555	0.8918	0.7447	0.5739	0.6675
	multi ant (M)	1,000	0.8320	0.8009	0.5609	0.7682	0.8188	0.7562
	multi ant (L)	10,000	0.8582	0.9519	**0.9304**	0.8734	0.8592	0.8946
	single ant	10,000	0.9354	0.9391	0.8993	0.9693	0.8225	0.9131
mixed	sb1	10,100	0.9894	0.9792	0.9194	**0.9828**	0.9214	**0.9507**
	sb5	10,500	0.9793	0.9348	0.8366	0.9546	0.8892	0.9038
	bs1000	11,000	0.9989	0.9541	0.7641	0.9529	0.9203	0.8979
	bs100	2,000	1.0000	0.9719	0.5331	0.9511	**0.9256**	0.8454
	bs10	1,1000	1.0000	0.9829	0.8442	0.9392	0.8773	0.9109

[1] Using five-fold cross-validation, 20% of the samples are withheld during training.

[2] Excluding AP from within-domain case

[3] Benchmark: reporting mAP across all cases, network has seen samples from every domain

[4] Augmented: background removed, replaced by random images pooled from ImageNet

Table 6 Case-specific tracking performance (MOTA) of YOLOv4 networks within OmniTrax (in *Atta*). All tracking was performed with a detection threshold of 0.5, a network input size of 1088 x 1088 and default OmniTrax settings. The best performing model for each unseen case is highlighted

data type	dataset	n real samples[1]	base	bright	dark	noisy	all (unseen)[2]
real	all[3]	6,000	0.996	0.997	0.998	0.997	0.997
real	base	1,000	0.996	**0.933**	0.795	0.541	0.756
	bright	1,000	0.870	0.996	0 [4]	0 [4]	0.290
	dark	1,000	0.807	0.577	0.997	0.608	0.664
	noisy	1,000	0.877	0.850	0.786	0.997	0.838
synthetic	single ant	0	**0.901**	0.859	0.945	0.821	0.882
mixed[5]	sb1	100	0.950	0.907	**0.947**	0.833	0.896
	sb5	500	0.968	0.916	0.926	**0.854**	**0.899**

[1] Using five-fold cross-validation, 20% of the samples are withheld during training.

[2] Excluding MOTA from within-domain case.

[3] Benchmark: reporting MOTA across all cases, network has seen samples from every domain

[4] True MOTA is negative given excessive number of false positives (FP)

[5] Mixed data includes 10,000 synthetic samples and the denoted number of hand-annotated samples of the base case.

Pose-estimation datasets

Table 7 Benchmark pose-estimation datasets

dataset	samples[1]	generator settings
top-down (platform)	179	camera = FLIR Blackfly, 18 mm lens<br>fps = 55<br>shutter speed = 1/200 s<br>image size (px) = 2048 x 1536<br>individuals = 1<br>notes: well exposed, no motion blur, static camera
left view (platform)	345	camera = FLIR Blackfly, 18 mm lens<br>fps = 55<br>shutter speed = 1/200 s<br>image size (px) = 2048 x 1536<br>individuals = 1<br>notes: well exposed, no motion blur, static camera
right view (platform)	381	camera = FLIR Blackfly, 18 mm lens<br>fps = 55<br>shutter speed = 1/200 s<br>image size (px) = 2048 x 1536<br>individuals = 1<br>notes: well exposed, no motion blur, static camera
all (platform)	805	camera = FLIR Blackfly, 18 mm lens<br>fps = 55<br>shutter speed = 1/200 s<br>image size (px) = 2048 x 1536<br>individuals = 1<br>notes: combining all platform views
handheld	200	camera = Huawei P20 Pro, Leica<br>fps = 25<br>shutter speed = 1/50 s<br>image size (px) = 1440 x 720 to 1920 x 1080<br>individuals = 1<br>notes: handheld phone camera, frequent occlusions, camera shake, harsh shadows

[1] Using five-fold cross-validation, 20% of the samples are withheld during training.

Table 8 Synthetic pose-estimation datasets

dataset	samples[1]	generator settings
single stick	10,000	individuals = 1<br>scale variation = 70<br>subject meshes = 1<br>image size (px) = 2048 x 2048<br>compression rand. = True<br>new terrain every = 10<br>new scatterers every = 5<br>new subject placement every = 1
multi stick	10,000	individuals = 5<br>scale variation = 70<br>subject meshes = 1<br>image size (px) = 2048 x 2048<br>compression rand. = True<br>new terrain every = 10<br>new scatterers every = 5<br>new subject placement every = 1

[1] Using five-fold cross-validation, 20% of the samples are withheld during training.

Table 9 Augmented / mixed *Sungaya* pose datasets

dataset	samples[1]	recording settings
handheld (refine)<br>12.5% real<br>25% real<br>50% real<br>100% real	 10,000 + 25<br>10,000 + 50<br>10,000 + 100<br>10,000 + 200	notes: combining the (synthetic) multi-ant dataset with the (real) base dataset in various ratios
platform (refine)<br>10 samples per camera	10,000 + 50	notes: combining the (synthetic) multi-ant dataset with the (real) base dataset in various ratios

[1] Using five-fold cross-validation, 20% of the samples are withheld during training.

Table 10 Pose-estimation performance (mean pixel error $\Delta\bar{y}$ and mean relative percentage error $\delta\bar{y}$) of DLC with ResNet101 backbone (in *Sungaya*, **platform data**). All metrics reported on withheld data only, with a 60% confidence threshold (default). Using five-fold cross-validation, 20% of the samples are withheld during training. As a rule of thumb, a mean relative error of $\delta\bar{y} \geq 10\%$ is unlikely to be suitable for pose-estimation in the given scenario.

	dataset	right view		left view		top-down		all views	
	body length	473.4 px		418.2 px		497.8 px		463.1 px	
data type	metric	$\Delta\bar{y}$	$\delta\bar{y}$	$\Delta\bar{y}$	$\delta\bar{y}$	$\Delta\bar{y}$	$\delta\bar{y}$	$\Delta\bar{y}$	$\delta\bar{y}$
real	all views	33.19	7.01	74.04	17.71	26.81	5.38	50.47	10.90
real	right view	13.90	2.94	342.49	81.90	49.31	9.91	135.23	31.58
	left view	326.62	69.00	14.82	3.54	351.32	70.57	230.92	47.70
	top-down	58.24	12.30	361.20	86.37	11.47	2.30	143.64	33.66
synthetic	single stick[1]	48.45	10.24	84.37	20.17	17.91	3.60	27.28	5.89
mixed	refine (10ea)[2]	**30.49**	**6.44**	**51.28**	**12.26**	**25.43**	**5.11**	**37.69**	**8.14**

[1] $\leq 50\%$ of the reported key-points were above threshold, leading to discarded low-confidence key-points.

[2] Fine-tuning the network trained on synthetic data (single stick) with 10 hand-annotated images from each of the 5 camera views.

Table 11 Pose-estimation performance (mean pixel error $\Delta\bar{y}$ and mean relative percentage error $\delta\bar{y}$) of DLC with ResNet101 backbone (in *Sungaya*, **handheld data**). All metrics reported on withheld data only, with a 60% confidence threshold (default). Using five-fold cross-validation, 20% of the samples are withheld during training. As a rule of thumb, a mean relative error of $\delta\bar{y} \geq 10\%$ is unlikely to be suitable for pose-estimation in the given scenario.

		dataset	handheld	
		body length	149.9 px	
data type	n real samples	metric	$\Delta\bar{y}$	$\delta\bar{y}$
real	200	full handheld	5.86	3.91
synthetic	0	single stick[1]	9.37	6.25
mixed[2]	25	12.5% real	9.72	6.48
	50	25% real	7.54	5.03
	100	50% real	5.32	3.55
	200	100% real	**5.16**	**3.44**

[1] $\leq 50\%$ of the reported key-points were above threshold, leading to discarded low-confidence key-points.

[2] Fine-tuning the network trained on synthetic data (single stick) with the reported number of randomly sampled hand-annotated frames of the **handheld** dataset.

Semantic segmentation datasets

Table 12 Synthetic pose-estimation datasets

dataset	samples[1]	generator settings
Leptoglossus (default)	10,000	individuals = 20<br>scale variation = 33<br>subject meshes = 1<br>image size (px) = 2048 x 2048<br>compression rand. = True<br>new terrain every = 10<br>new scatterers every = 5<br>new subject placement every = 1<br>notes: using default scatterers
Leptoglossus (plants)	10,000	individuals = 20<br>scale variation = 33<br>subject meshes = 1<br>image size (px) = 2048 x 2048<br>compression rand. = True<br>new terrain every = 10<br>new scatterers every = 5<br>new subject placement every = 1<br>notes: adding asset scatterers using plant assets from Quixel library including 20 grass and 11 fern and shrub assets

[1]Using five-fold cross-validation, 20% of the samples are withheld during training.

Insect rigging convention

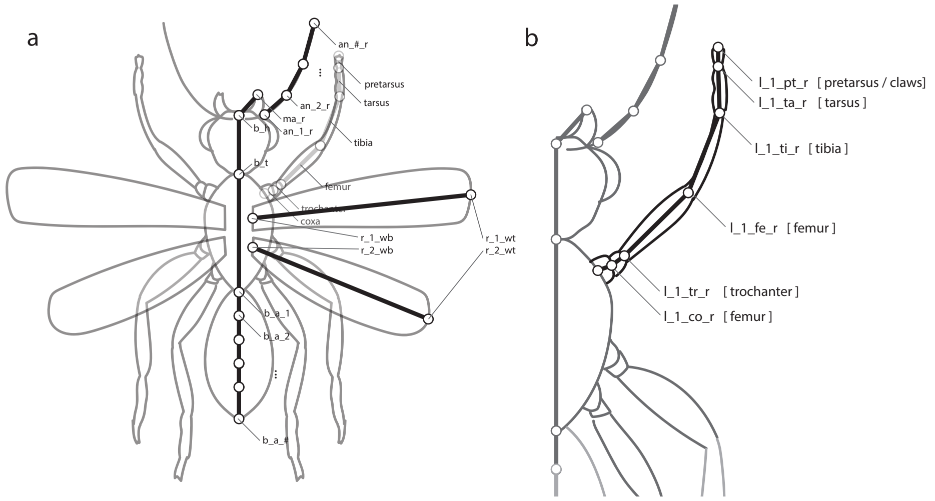

Supplementary Figure 1 A standardised rig forms the basis of insect model actuation within the *replicAnt* pipeline. (a) As all insects follow the same fundamental body layout, we have created a standardised rig that is used to actuate all insect models inside the *replicAnt* generator pipeline. The rig can be extended to provide further articulation, such as additional segmentation or additional appendages, e.g., articulated proboscis. (b) The employed Inverse Kinematics Solver (IKS) within *replicAnt* expects the depicted rigging and naming convention to use our provided animation blueprints. We provide additional documentation and a template Blender project file on out GitHub page to simplify the rigging process for new users. (https://github.com/evo-biomech/replicAnt)

Supplementary Note 1

Armature An Armature is a collection of virtual bones and joints which define how the the mesh deforms as pose changes. The process of assigning an armature to a mesh is referred to as rigging.

Asset An element from a library of pre-existing meshes placed in the generated 3D environment.

Buffer temporarily stored visual information, in this context generated 2D texture maps.

Decal A generated 2D material, projected onto multiple assets in the 3D environment; albedo, alpha, roughness, metalness, and normal maps tie the appearance of distinct assets together.

Iteration An iteration describes a single step of a procedurally generated dataset. Its output is a collection of associated passes and labels.

Label A label contains the information found in a corresponding image sample in ordered text form, including bounding boxes, IDs, as well as 2D and 3D joint locations.

Mesh A 3D object defined by polygons.

Network Various deep neural networks for computer vision applications such as classification, detection, tracking, 2D and 3D pose estimation, or semantic segmentation.

Parser A program written to interpret a given input and translate it into a different format. We use parsers extensively to convert generated data (image passes and labels) into formats readable by various deep learning frameworks.

Pass A pass (or render pass) is a 2D image containing either the rendered camera view, segmentation data, depth data, or normal data in object or world space.

Subject A subject is an imported, rigged mesh which is part of the generated population, i.e., a group of mesh-es for which we export annotated data.

Texture maps :

- **Albedo** The albedo or base colour map contains the colour information of a material in Physically Based Rendering (PRB) workflows
- **Alpha** The alpha map controls the opacity of specific areas of the material.
- **Depth** The depth or displacement map is a single channel texture which describes (large) height changes in the mesh topology. These maps are usually combined with a weight and a bias to influence the magnitude and base height of the resulting displacement.

Metalness There are two types of reflection models used in PBR materials: dielectric and conductive. The metalness map functions as a mask to locally blend between the two.

normal The normal map contains small scale surface detail information to increase the perceived complexity of the mesh's appearance without increasing polygon count. Normal maps consist of three colour channels to encode local position information of the surface normal in X, Y, and Z respectively (see also displacement maps).

Roughness The roughness map controls the reflectivity of a material, i.e.it controls transitions between diffuse and glossy appearance.
