# Supplementary material for: replicAnt: a pipeline for generating annotated images of animals in complex environments using Unreal Engine
Source: Nat Commun. 2023 Nov 8;14:7195. doi: 10.1038/s41467-023-42898-9 (PMC10632501; doi:10.1038/s41467-023-42898-9)
Supplement: Supplementary file 3 — Description of Additional Supplementary Files [file 41467_2023_42898_MOESM3_ESM.pdf]

Description of Additional Supplementary Files

Supplementary Video 1
Description: Multi-animal tracking demo, showcasing models trained on synthetic data produced with replicAnt loaded into OmniTrax to track the position of freely moving animals in a variety of laboratory and field settings.

Supplementary Video 2
Description: Multi-animal tracking and pose estimation demo, using a YOLOv4 detector trained exclusively on synthetic data and a DeepLabCut pose-estimator trained on mixed synthetic and real samples.

Supplementary Video 3
Description: Automatically tracked video of Atta cephalotes recorded in the field by Samuel T. Fabian, processed with OmniTrax, using YOLOv4 detectors trained exclusively on synthetic samples, generated with replicAnt.

Supplementary Video 4
Description: Automatically tracked video of *Atta cephalotes* with frequent lighting changes, recorded in the field by Samuel T. Fabian, processed with OmniTrax, using YOLOv4 detectors trained exclusively on synthetic samples, generated with replicAnt.

Supplementary Video 5
Description: Pose estimation performance comparison of a DeepLabCut single animal pose estimation network trained on >800 real hand-annotated images of the same species (*Sungaya inexpectata*) in a different recording setup VS the same network trained exclusively on synthetic samples, generated with replicAnt.

Supplementary Video 6
Description: Pose estimation performance demo showcasing how providing a few hand annotated samples to a pose estimator pre- trained on synthetic samples leads to robust performance with minimal manual effort.

Supplementary Video 7
Description: Multi-animal tracking and pose estimation demo within OmniTrax on a large number of freely moving Atta vollenweideri, using a YOLOv4 detector trained exclusively on synthetic data and a DeepLabCut pose-estimator trained on mixed synthetic and real samples.
