# Supplementary material for: replicAnt: a pipeline for generating annotated images of animals in complex environments using Unreal Engine
Source: Nat Commun. 2023 Nov 8;14:7195. doi: 10.1038/s41467-023-42898-9 (PMC10632501; doi:10.1038/s41467-023-42898-9)
Supplement: Supplementary file 11 — Reporting Summary [file 41467_2023_42898_MOESM11_ESM.pdf]

nature portfolio

Corresponding author(s): Fabian Plum
David Labonte

Last updated by author(s): 26/09/2023

# Reporting Summary

Nature Portfolio wishes to improve the reproducibility of the work that we publish. This form provides structure for consistency and transparency in reporting. For further information on Nature Portfolio policies, see our Editorial Policies and the Editorial Policy Checklist.

## Statistics

For all statistical analyses, confirm that the following items are present in the figure legend, table legend, main text, or Methods section.

| n/a | Confirmed | |
|---|---|---|
| ☐ | ☒ | The exact sample size (*n*) for each experimental group/condition, given as a discrete number and unit of measurement |
| ☐ | ☒ | A statement on whether measurements were taken from distinct samples or whether the same sample was measured repeatedly |
| ☒ | ☐ | The statistical test(s) used AND whether they are one- or two-sided<br>*Only common tests should be described solely by name; describe more complex techniques in the Methods section.* |
| ☒ | ☐ | A description of all covariates tested |
| ☒ | ☐ | A description of any assumptions or corrections, such as tests of normality and adjustment for multiple comparisons |
| ☐ | ☒ | A full description of the statistical parameters including central tendency (e.g. means) or other basic estimates (e.g. regression coefficient) AND variation (e.g. standard deviation) or associated estimates of uncertainty (e.g. confidence intervals) |
| ☒ | ☐ | For null hypothesis testing, the test statistic (e.g. *F*, *t*, *r*) with confidence intervals, effect sizes, degrees of freedom and *P* value noted<br>*Give P values as exact values whenever suitable.* |
| ☒ | ☐ | For Bayesian analysis, information on the choice of priors and Markov chain Monte Carlo settings |
| ☒ | ☐ | For hierarchical and complex designs, identification of the appropriate level for tests and full reporting of outcomes |
| ☒ | ☐ | Estimates of effect sizes (e.g. Cohen's *d*, Pearson's *r*), indicating how they were calculated |

*Our web collection on statistics for biologists contains articles on many of the points above.*

## Software and code

Policy information about availability of computer code

| | |
|---|---|
| Data collection | SpinView v2.7.0.128, depthai v0.4.0.0, OmniTrax v0.2.1 (https://github.com/FabianPlum/OmniTrax), replicAnt v1.0.0 (https://github.com/evo-biomech/replicAnt), scAnt (https://github.com/evo-biomech/scAnt) |
| Data analysis | replicAnt v1.0.0 (https://github.com/evo-biomech/replicAnt), cudatoolkit (10.0.130), cudnn (7.6.5), Yolov4 (https://github.com/AlexeyAB/darknet), deeplabcut (2.2.1.1) ,deeplabcut-live (1.0.2), OmniTrax v0.2.1 (https://github.com/FabianPlum/OmniTrax), MMSegmentation (0.29.1), h5py (2.10.0), imutils (0.5.3), jupyter (1.0.0), json5 (0.9.5), keras (2.3.1), matplotlib (3.2.2), opencv-python (4.5.3.56), pandas (1.1.2), scikit-image (0.16.2), scikit-learn (0.23.1), scipy (1.5.0), tensorflow (1.14.0), Blender (v2.92 & v3.1), Unreal Engine (5.0.3) |

For manuscripts utilizing custom algorithms or software that are central to the research but not yet described in published literature, software must be made available to editors and reviewers. We strongly encourage code deposition in a community repository (e.g. GitHub). See the Nature Portfolio guidelines for submitting code & software for further information.

## Data

Policy information about availability of data

All manuscripts must include a data availability statement. This statement should provide the following information, where applicable:

- Accession codes, unique identifiers, or web links for publicly available datasets
- A description of any restrictions on data availability
- For clinical datasets or third party data, please ensure that the statement adheres to our policy

All datasets, both generated and real, additional SI, and the best performing networks are hosted via Zenodo:
- 3D Models https://zenodo.org/record/7849059, DOI : 10.5281/zenodo.7849059
- Detection and Tracking Datasets and Trained networks https://zenodo.org/record/7849417, DOI : 10.5281/zenodo.7849417
- Pose-Estimation Datasets and Trained networks https://zenodo.org/record/7849596, DOI : 10.5281/zenodo.7849596
- Semantic And Instance Segmentation Datasets and Trained networks https://zenodo.org/record/7849570, DOI : 10.5281/zenodo.7849570

## Research involving human participants, their data, or biological material

Policy information about studies with human participants or human data. See also policy information about sex, gender (identity/presentation), and sexual orientation and race, ethnicity and racism.

| | |
|---|---|
| Reporting on sex and gender | N/A |
| Reporting on race, ethnicity, or other socially relevant groupings | N/A |
| Population characteristics | N/A |
| Recruitment | N/A |
| Ethics oversight | N/A |

Note that full information on the approval of the study protocol must also be provided in the manuscript.

# Field-specific reporting

Please select the one below that is the best fit for your research. If you are not sure, read the appropriate sections before making your selection.

☐ Life sciences ☐ Behavioural & social sciences ☒ Ecological, evolutionary & environmental sciences

For a reference copy of the document with all sections, see nature.com/documents/nr-reporting-summary-flat.pdf

# Ecological, evolutionary & environmental sciences study design

All studies must disclose on these points even when the disclosure is negative.

| | |
|---|---|
| Study description | To demonstrate the inference performance of deep neural networks trained on both hand-annotated and synthetically generated data for various computer vision applications common to animal behavioural research, we collected a number of video recordings of freely moving animals. We recorded video footage of individual walking Sungaya inexpectata specimens, as well as large groups of Atta vollenweideri under laboratory conditions. We additionally recorded a group of freely moving Gnathamitermes spec. in the field to provide an example of an unpredictable recording environment. As the focus of this study lies in the quantification of inference performance, no specific randomisation of in the selection of study animals was applied. |
| Research sample | Individual Sungaya inexpectata from a laboratory colony, walking on a multi-camera recording plane. Animals were either selected at random. Groups of Atta vollenweideri, part of a laboratory colony, recorded as they traverse from their nest to their foraging site. A group of Gnathamitermes spec. recorded close to the nest entrance in the desert of Maricopa County. Both A. vollenweideri and G. spec. were recorded without interfering with the animals and no specific individuals were selected. |
| Sampling strategy | Sample size was dependent on the dataset, and summarised in the supplementary information. All synthetically generated datasets contain 10,000 (or more) annotated samples and real (hand-labeled) datasets each contain >200 to 6000 samples. In case of "mixed", or "refined" training approaches, using both synthetic and real data, the exact numbers and splits are documented in the supplementary information. |
| Data collection | Hendrik Beck recorded all videos of Sungaya inexpectata specimens with a tiltable, planar, synchronised 5 camera setup. Fabian Plum and Natalie Imirzian recorded all Atta vollenweideri videos with a machine vision camera positioned above an acrylic container connected to the foraging trail of the colony. Fabian Plum recorded the video (and still images for qualitative examples) of Gnathamitermes spec. with a DSLR camera on a tripod. |

| | |
|---|---|
| Timing and spatial scale | N/A |
| Data exclusions | No data were excluded |
| Reproducibility | For all reported experiments 5-fold cross validation for held-out and included samples was used, the exception being qualitative results reported in the image segmentation examples. Training of image segmentation networks with various species confirmed the reproducibility of the reported qualitative results. |
| Randomization | Training/testing dataset splits were generated randomly without replacement and kept constant across all experiments using the same dataset. In case of synthetic data generation, the required models, settings and randomisation seeds are reported for reproduction. |
| Blinding | N/A |

Did the study involve field work? ☒ Yes ☐ No

## Field work, collection and transport

| | |
|---|---|
| Field conditions | The field recordings of Gnathamitermes spec. were taken in dry and slightly overcast weather conditions. |
| Location | Desert of Maricopa County, approximately at (33.725, -111.663) |
| Access & import/export | N/A |
| Disturbance | Animals were recorded in their natural habitat without any direct interaction. |

# Reporting for specific materials, systems and methods

We require information from authors about some types of materials, experimental systems and methods used in many studies. Here, indicate whether each material, system or method listed is relevant to your study. If you are not sure if a list item applies to your research, read the appropriate section before selecting a response.

### Materials & experimental systems

| n/a | Involved in the study | |
|---|---|---|
| ☒ | ☐ | Antibodies |
| ☒ | ☐ | Eukaryotic cell lines |
| ☒ | ☐ | Palaeontology and archaeology |
| ☐ | ☒ | Animals and other organisms |
| ☒ | ☐ | Clinical data |
| ☒ | ☐ | Dual use research of concern |
| ☒ | ☐ | Plants |

### Methods

| n/a | Involved in the study | |
|---|---|---|
| ☒ | ☐ | ChIP-seq |
| ☒ | ☐ | Flow cytometry |
| ☒ | ☐ | MRI-based neuroimaging |

## Animals and other research organisms

Policy information about studies involving animals; ARRIVE guidelines recommended for reporting animal research, and Sex and Gender in Research

| | |
|---|---|
| Laboratory animals | Atta vollenweideri, Sungaya inexpectata |
| Wild animals | Gnathamitermes spec., recorded in the field, no animals were captured. |
| Reporting on sex | n/a |
| Field-collected samples | n/a |
| Ethics oversight | All research conducted in the European Union involving vertebrates, cyclostomes or cephalopods must comply with the European Directive 63/2010/EU. A. vollenweideri and S. inexpectata neither fall under this directive, nor are they considered protected species under the Convention on International Trade in Endangered Species (CITES), no specific permits are required. All proposed experiments are designed to minimise animal suffering and are unlikely to cause harm. |

Note that full information on the approval of the study protocol must also be provided in the manuscript.
